# Recombinant β-Carotene Production by *Yarrowia lipolytica* – Assessing the Potential of Micro-Scale Fermentation Analysis in Cell Factory Design and Bioreaction Optimization

**DOI:** 10.3389/fbioe.2020.00029

**Published:** 2020-02-13

**Authors:** Irene Hjorth Jacobsen, Rodrigo Ledesma-Amaro, José Luis Martinez

**Affiliations:** ^1^Department of Biotechnology and Biomedicine, Section for Synthetic Biology, Technical University of Denmark, Kongens Lyngby, Denmark; ^2^Department of Bioengineering, Faculty of Engineering, Imperial College London, London, United Kingdom

**Keywords:** micro-fermentation, β-carotene, *Yarrowia lipolytica*, yeast cell factories, metabolic engineering, fermentation, media optimization

## Abstract

The production of β-carotene has become increasingly interesting within the biotechnological industry due to a rising demand for safer and more natural colorants, nutritional supplements, and antioxidants. A recent study has described the potential of *Yarrowia lipolytica* as a β-carotene-producing cell factory, reporting the highest titer of recombinant β-carotene produced to date. Finding the best conditions to maximize production and scaling up the process to full scale, a costly and time-consuming process, it is often a bottleneck in biotechnology. In this work, we explored the benefits of using micro-fermentation equipment to significantly reduce the time spent on design and optimization of bioreaction conditions, especially in the early stages of process development. In this proof-of-concept study, a β-carotene producing *Y. lipolytica* strain was tested in micro-fermentations partly to assess the robustness of the cell factory design and partly to perform media optimization. The medium optimization led us to an improvement of up to 50% in the yield of β-carotene production in the best of the conditions. Overall, the micro-fermentation system had a high degree of reliability in all tests.

## Introduction

The orange-colored tetraterpenoid β-carotene, is naturally produced in varying concentrations by several plants and other eukaryotes, including carrot (*Daucus carota* subsp. *sativus*) and the micro-algae *Dunaliella salina* (Cuttriss et al., 2011). In plants, humans, and other animals, the compound provides antioxidant properties whereby Reactive Oxygen Species (ROS) are eliminated (Russell and Paiva, 1999; Cuttriss et al., 2011; Bogacz-Radomska and Harasym, 2018). Furthermore, β-carotene is beneficial to humans in the form of vitamin A, which is involved in multiple processes such as (i) maintenance of the chromophores in the eye, (ii) prevention of blindness and night-blindness, (iii) ensuring reproductive efficiency, and (iiii) maintaining a healthy growth (Ribeiro et al., 2011). Another major application for β-carotene is in the food industry where it is used as a natural pigment for a variety of foods, including but not limited to sweets, non-alcoholic beverages, and cheese (Mortensen, 2006; Bogacz-Radomska and Harasym, 2018).

The interest for β-carotene as a colorant and as a supplement is increasing, thus the market has been predicted to rise from USD 432.2 million in 2015 to USD 600 million by 2024 (Grand View Research, 2016; Larroude et al., 2017). Commercially β-carotene is produced through one of three routes: (i) chemical extraction in which the β-carotene is extracted from fruits or vegetables, disadvantages including high cost, seasonal variations, and low productivities (Bogacz-Radomska and Harasym, 2018); (ii) chemical synthesis from acetone, butadiene, di-aldehyde, and methanol. Some negative effects, including cancer, have been associated with the ingestion of high doses of synthetic β-carotene in some population groups (Hartman et al., 1995; Woutersen et al., 1999; Black, 2004; Ribeiro et al., 2011; Bogacz-Radomska and Harasym, 2018); and (iii) microbial biosynthesis, where the main producer is *D. salina* with reported productivities of 0.001–0.05 g/L per day (Guedes et al., 2011; Ribeiro et al., 2011; Bogacz-Radomska and Harasym, 2018). As of the beginning of the current decade, 98% of all commercial β-carotene was produced either using physicochemical synthesis or chemical synthesis, whilst the remaining 2% had biological origin (Ribeiro et al., 2011).

**FIGURE 1:**
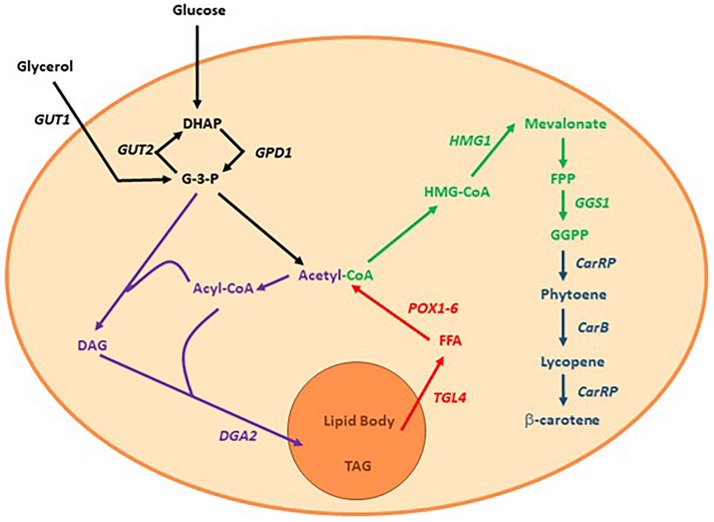
Simplified view of the lipogenesis and mevalonate pathway in *Y. lipolytica.* Purple: the lipogenesis using glyceraldehyde-3-phosphate and acetyl-CoA as building blocks, Red: mobilization and β-oxidation from lipid bodies to acetyl-CoA, Green: mevalonate pathway, Dark blue: heterologous genes introduced by Larroude et al. (2017) DHAP, Dihydroxyacetone phosphate; G-3-P, Glyceraldehyde-3-phosphate; DAG, Diacylglycerol; TAG, Triacylglycerol; FFA, Free fatty acid; HMG-CoA, 3-hydroxy-3-methylglutaryl-co-enzyme A; GUT2, Glycerol-3-phosphate dehydrogenase 2; GUT1, Glycerol kinase 1; DGA2, Diacylglycerol acyl transferase; TGL4, Triacylglycerol lipase 4; POX1-6, Fatty-acyl coenzyme A oxidase; GGS1, Geranylgeranyl pyrophosphate synthase; CarRP, Phytoene synthase; CarB, Phytoene dehydrogenase.

Nevertheless, the high demand has brought a new player to the field: the recombinant production of β-carotene. Safety studies of recombinant β-carotene found no toxicity or deviations compared to commercially available β-carotene (Grenfell-Lee et al., 2014). Thus, several organisms have been engineered to become β-carotene producing cell factories, e.g., *Yarrowia lipolytica* has been chosen as the production workhorse by multiple research teams both in academia and industry (Zhu and Jackson, 2015). Even though *Y. lipolytica* does not naturally produce β-carotene, it is well known for its ability to produce and accumulate lipids; lipids and β-carotene share the same precursor, acetyl-CoA, and the genes and metabolic pathways required for producing lipids and β-carotene are both known (Beopoulos et al., 2008; Harzevili, 2014; Larroude et al., 2017). Recently, Larroude et al. (2017) engineered a recombinant lipid-overproducing strain of *Y. lipolytica* in order to produce β-carotene. The enhanced lipogenesis lead to an increase in the metabolic flux toward the mevalonate pathway, as depicted in Figure 1. This recombinant strain, namely *Y. lipolytica* ob-CHC^TEF^C^TEF^, produced 6.5 g β-carotene/L in a single fed-batch experiment, which corresponded to 90 mg β-carotene/gDW (Larroude et al., 2017). To our knowledge, this is the best production strain reported to date.

In recent years, high-throughput methods have been established and verified for strain construction (Festa et al., 2013; Jensen et al., 2014) and high-throughput systems like the BioLector^®^ have been developed to better characterize and screen strain libraries. To this respect, the BioLector^®^ has the capacity to run 48 fully controlled and scalable cultivations in a deep-well micro-titer plate. This device can moreover measure cell density, fluorescence, NAD(P)H, riboflavin, pH, and dissolved oxygen. Additionally, the design of the cultivation plate allows for oxygen transfer rates similar to the ones reported in bench-top bioreactor systems (Rohe et al., 2012; Back et al., 2016).

Our present work has two major focus points: (1) investigating the efficacy of parameters involved in β-carotene production by *Y. lipolytica* ob-CHC^TEF^C^TEF^ in a batch fermentation setup, at both micro-scale and lab-scale, and (2) proving that the use of micro-fermentation systems for analysis and early bioreaction design, can decrease the time spent on strain development and testing with a high degree of reliability. Multiple strategies for enhancing lipid production and lipid accumulation in *Yarrowia* have been described in literature, such as depletion of nitrogen offsets lipid accumulation, which is achieved using a high carbon-to-nitrogen ratio (C/N ratio) ideally in a continuous cultivation (Gropoşila-Constantinescu et al., 2015; Kavšcek et al., 2015). It has also been described that lipid production in *Yarrowia* is favored at pH 5–6.5 (Beopoulos et al., 2008; Kuttiraja et al., 2017). Finally, the addition of the monounsaturated oleic acid as a co-substrate has also been reported to promote lipid accumulation (Beopoulos et al., 2008). Higher production of β-carotene has been reported in lipid accumulating strains (Larroude et al., 2017), showing a clear link between these two pathways. We hypothesized that the abovementioned conditions would maximize production.

In this work, we first optimized culture conditions using micro-fermentors in order to maximize the production of β-carotene in batch fermentations. In terms of proving and predicting scalability of micro-fermentations in cell factory design, several of the experiments were further scaled-up to 1L bioreactors. The experiments in 1L scale included an optimal medium design, a near optimal medium design, and a non-optimal medium design.

## Materials and Methods

### Strain and Culture Conditions

The strain *Y. lipolytica* ob-CHC^TEF^C^TEF^, provided by Rodrigo Ledesma-Amaro, was constructed in 2017 by Larroude et al. (2017). The strain is based on a lipid-overproducer derived from *Y. lipolytica* w29 (ATCC20460) using a gene cassette, “car-cassette,” from Microbia Inc. containing geranylgeranyl pyrophosphate synthase (*GGS1*), phytoene synthase/lycopene cyclase (*CarRP*), and phytoene dehydrogenase (*CarB*) under three different promotors, and lastly a gene cassette containing hydroxymethylglutaryl-CoA reductase (*HMG1*). The construction itself was carried out using Golden Gate Assembly. Four rounds of transformations were carried out: firstly, the car-cassette was transformed into the overproducing strain; secondly, the *HMG1*-casette was inserted; thirdly, an optimized car-cassette (car^TEF^-cassette) was inserted; and lastly a second car^TEF^-cassette was inserted. The final strain, *Y. lipolytica* ob-CHC^TEF^C^TEF^, had therefore three car-cassettes and one *HMG1*-casette inserted (Larroude et al., 2017). The organism was maintained on solid 2% YPD plates (10 g/L yeast extract, 20 g/L peptone, 20 g/L glucose). Pre-cultures were prepared with media matching the medium used afterward in each individual culture condition described. The pre-cultures were incubated at 150 rpm and at 28°C, during approximately 30 h prior to inoculation.

For the micro-scale fermentations, three types of experiments were carried out: pH, carbon-nitrogen ratio (C/N), and oleic acid addition. For pH and C/N, glucose and glycerol were both tested as carbon sources. For all conditions tested (pH, C/N, and oleic acid experiments) Delft medium was used as the basis, containing: (NH_4_)2SO_4_: 5 g/L, KH_2_PO_4_: 3 g/L, MgSO_4_^∗^7H_2_O: 0.5 g/L, 1mL/L trace metal solution (contents per liter; FeSO_4_^∗^7H_2_O: 3 g/L, ZnSO_4_^∗^7H_2_O: 4.5 g/L, CaCl_2_^∗^6H_2_O: 4.5 g/L, MnCl_2_^∗^2H_2_O: 0,84 g/L, CoCl_2_^∗^6H_2_O: 0,3 g/L, CuSO_4_^∗^5H_2_O: 0,3 g/L, Na_2_MoO_4_^∗^2H_2_O: 0,4 g/L, H_3_BO_3_: 1 g/L, KI: 0,1 g/L, Na_2_EDTA: 15 g/L) and 1 mL/L vitamin solution (contents per liter; d-biotin: 0.05 g/L, Ca-pantothenat: 1 g/L, thiamine-HCl: 1 g/L, pyridoxine-HCl, nicotinic acid: 1 g/L, p-aminobenzoic acid: 0.2 g/L, m-inositol: 25 g/L). The C/N-ratios were varied between 0 and 18.25 for glucose and between 0 and 7 for glycerol, and were based on the number of carbon atoms divided by the number of nitrogen atoms. In practice, the following calculations were carried out (exemplified with glucose): (1) the number of moles of glucose in the standard medium (20 g/L glucose) was calculated, (2) the difference in molecular weights was accounted for, by dividing the MW of pure carbon with the MW of glucose, and (3) the number of moles of carbon in the standard medium (20 g/L glucose) was calculated based on the results from the two previous steps and finally normalized to one atom. The C/N of standard CBS-glucose medium was calculated to 1.83 and thus C/N 3.65 is equal to two carbon atoms per one nitrogen atom. These calculations were done for glucose, glycerol, and ammonium sulfate. In order to achieve C/N values lower than the standard, the amount of ammonium sulfate was increased. Due to the difference in elemental composition of glucose and glycerol, C/N 7.3 for glucose corresponded to 80 g/L whereas C/N 7 for glycerol corresponded to the 20 g/L present in the standard medium.

The concentration of the carbon-source was kept constant at 20 g/L for pH and oleic acid experiments. Oleic acid was added in the form of olive oil, which contained 72% pure oleic acid, in concentrations of 1, 2, 3, and 4%.

For all experiments, 20.4 g/L of potassium hydrogen phthalate was used for pH buffering. The micro-fermentations were carried out using a BioLector^®^ I system (m2p Labs, Germany) with a 48 well FlowerPlate^®^ (MTP-48-B, m2p Labs, Germany). The following settings were programmed: filter; biomass (gain 10), humidity; on (>85% using ddH_2_O), temperature; 28°C, oxygen supply; 20.85% (atmospheric air), agitation speed; 1000 rpm. The total volume of each well was 1500 μL, initial OD_600_ was 0.01.

For lab-scale fermentations four different experiments with four different media compositions were carried out, as follows; CBS-gly C/N 7: Delft medium with 20 g/L of glycerol (as described above), pH: 5; CBS-glc C/N 7.3: Delft medium (as described above) with 80 g/L of glucose, pH: 5; CBS-opt + OA/CBS-opt%OA: (NH_4_)2SO_4_: 40 g/L, KH_2_PO_4_: 3 g/L, MgSO_4_^∗^7H_2_O: 0.5 g/L, glycerol: 20 g/L, vitamin solution 1 mL/L, trace metal solution 1 mL/L, pH 7; CBS-opt%OA had no further additives. CBS-opt + OA contained 1% oleic acid added as olive oil. The cultivations were performed in 1L BIOSTAT Q Plus bioreactors (Sartorius Stedim Biotech S.A, Germany) using the software MFCS (version 3.0, Sartorius AG, Germany) for process monitoring and data acquisition. Process parameters: pH was adjusted and further controlled with 2M H_2_SO_4_ and 2M KOH, temperature: 28°C, airflow: 1 vvm, agitation: 800 rpm. The initial OD_600_ = 0.1. Off-gas was analyzed by MS (Thermo Scientific, model: Prima Pro, United Kingdom) and monitored online throughout the run.

### Sample Analysis

For the lab-scale bioreactor experiments, the optical density was determined by spectrophotometry at 600 nm. The biomass concentration was determined as dry weight: biomass was filtered (pore size 0.45 μm) and dried in a microwave at 180 watt during 20 min, and the dry biomass was then weighted. Background corrected CO_2_ was determined by MS. The substrate consumption and exo-metabolites were determined by ion-exchange HPLC. Prior to the HPLC analysis, the samples were filtered (pore size 0.45 μm). The injection volume was 20 μL, the eluent was 5 mM H_2_SO_4_ and the flow rate was 0.6 mL/min. A Bio-Rad Aminex HPX-87H column (Hercules, CA, United States) was used and kept at 60°C. The exo-metabolites were quantified using a RID-detector.

For the micro-fermentations: light scattering units (LSU) were measured every 7.29 min and logged by the BioLector^®^. The biomass was determined from one representative replicate by dry weight analysis using the filtration method described above.

### β-Carotene Extraction and Quantification

Crude fermentation samples were freeze-dried before the solvent [50:50 v/v Heptane (Sigma Aldrich, Germany), Ethyl Acetate (Sigma Aldrich, Germany) with 0.01% Butylated Hydroxytoluene (Sigma Aldrich, Germany)] and the glass beads (710–1180 microns, Sigma Aldrich, St. Louis, MI, United States) were added. The tubes were then vortexed, ultrasonicated for 20 min, and centrifuged at 13,000 *g* for 5 min. The supernatant was transferred to new tubes and the solvent was evaporated under nitrogen in the dark. The extract was re-suspended in HPLC solvent [99.9% ethanol (Kemetyl, Køge, Denmark) and 0.01% Butylated Hydroxytoluene (Sigma Aldrich, Germany)].

Samples were analyzed by HPLC with a Kinetex^®^ C8 LC column with 2.6 μm silica particles (Phenomenex, CA, United States). Gradient mode was applied at a flow rate of 0.3 mL/min; solvent A was methanol/milli-Q water with 50 mM ammonium acetate (95%) and solvent B was methanol (5%). The pressure was set to minimum 60 bar. The gradient was set to 11 min. The column oven was kept at 60°C and the DAD was set to detect β-carotene at 450 nm. An external β-carotene standard containing 30 mg/L in HPLC solvent (Sigma Aldrich, Germany) was used for quantification. The standard curve had nine points that ranged from the lowest possible injection volume to saturation. For our samples, multiple injections were performed at different volumes. During the data analysis, only the data that fell within the linear range of the standard curve were considered, and then multiplied by the corresponding dilution factor. For all experiments the β-carotene concentration was determined for representative replicates only.

### Microfermentation Data Analysis

The recorded LSU data was corrected for outliers and the specific growth rate was determined from triplicates. Yields were determined from single data points using this formula (1). For yield-calculations it was assumed that all the added carbon-source had been consumed, as quantification was not possible due to the low volume. Oleic acid was considered a carbon source.

(1) $$Y_{i\,j}=\frac{C\,o\,n\,c.j\,\left[\frac{g}{L}\right]}{C\,o\,n\,c\,i.\left[\frac{g}{L}\right]}\;$$

### Bioreactor Data Analysis

Specific growth rates were calculated from triplicates based on three types of measurements: dry weight, OD_600_, and accumulated CO_2_. Specific growth rate from CO_2_ was calculated according to the method described by Knudsen et al. (2015). Yields were calculated using formula (1) for comparability.

## Results and Discussion

### High-Throughput Screening of Different Media Designs

**FIGURE 2:**
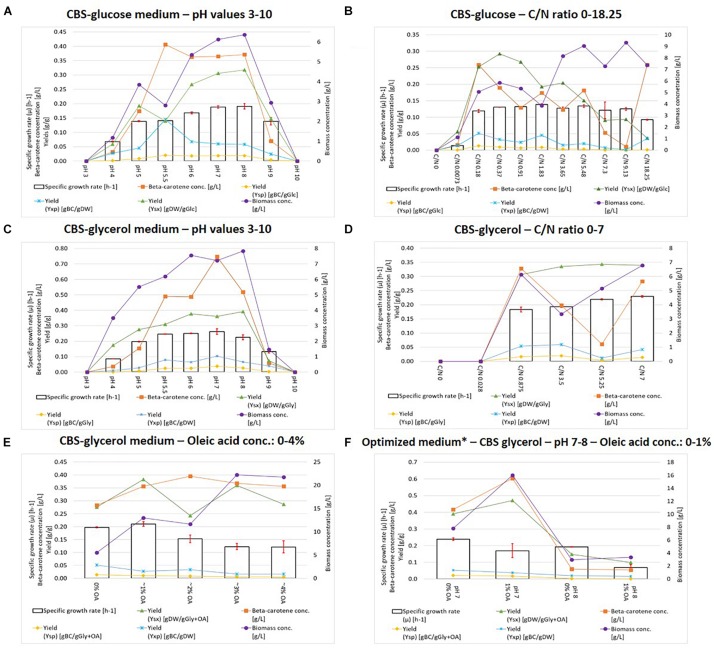
BioLector^®^ micro-fermentations. Top panel: cultivations with CBS glucose medium with varying pH values **(A)** and C/N ratios **(B)**. Middle panel: cultivations with CBS glycerol medium with varying pH values **(C)** and C/N ratios **(D)**. Lower panel: cultivations with CBS glucose medium with oleic acid addition **(E)** and optimized medium **(F)**. ^∗^CBS-gly-opt%OA/CBS-gly-opt + OA.

In order to establish the optimal batch fermentation conditions, micro-fermentations were carried out to assess how the pH, the C/N ratio, or the addition of oleic acid could influence the yield and/or productivity of β-carotene from two different carbon sources (glucose and glycerol), without drastically impacting the growth rate or the biomass yield. Figures 2A–E shows the results from the micro-fermentation runs. The strain performance in each medium was evaluated based on six optimization-parameters: specific growth rate (μ), final biomass concentration, the yield of biomass on substrate (Y_sx_), the final β-carotene concentration, the yield of β-carotene on substrate (Y_sp_), and the yield of β-carotene on biomass (Y_xp_). For both glucose and glycerol, no growth was observed at pH 3 or pH 10 in our experimental conditions. It is worth mentioning that growth at pH 3 had previously been reported for other *Yarrowia* strains different than the one used for this study, however, that was in chemostat and batch fermentations at higher glycerol concentrations than the 20 g/L tested in this study (Rakicka et al., 2017a, b). At pH 9 and pH 4 some growth, biomass, and β-carotene production were observed. The optima for all six optimization parameters were found near the neutral to slightly alkaline pH values (6–8). Very little variation was seen between pH 7 and pH 8, and therefore both values were chosen for the optimized media design.

In the glycerol-containing medium, the strain performed better than in the one containing glucose, based on the six measured optimization parameters. It has previously been reported by Workman et al. (2013) that *Yarrowia* has a higher capacity for glycerol uptake than glucose uptake, therefore our results are in accordance with these findings. Surprisingly, the optimal β-carotene production was not observed at pH 5–6.5 as expected but rather at pH 7–8. Due to the difference in elemental composition of glycerol and glucose, the mole-based C/N ratios differed. For glucose, C/N ratios between 0 and 18.25 (corresponding to 0 g/L glucose and 200 g/L glucose respectively) were used. The ratio of 1.83 corresponds to the standard 20 g/L of glucose. For glycerol, ratios between 0 and 7 (corresponding to 0 g/L glycerol and the standard 20 g/L glycerol respectively) were used. For both carbon sources the optimization parameters had their optima at the lower (below or around the standard) C/N ratios, as seen in Figures 2B,D. Only the final biomass concentration increased with increasing C/N, due to the abundance of carbon. The biomass yield (Y_sx_) decreased at higher C/N ratios for glucose, hence once again proving glycerol superior to glucose as carbon source. According to Figure 2D, the highest values for β-carotene concentration, Y_xp_, and Y_sp_, were observed for CBS-glycerol at C/N 0.875, which was chosen for the optimized media design.

High initial concentration of carbon-source has been reported to inhibit biomass formation in *Yarrowia*, which is seen for CBS-glc C/N 18.25 (Kuttiraja et al., 2016). The effect of lipid co-substrates on performance was further investigated by the addition of olive oil to the culture medium. According to Figure 2E, the optima for the six optimization parameters were observed at 0 or 1% of oleic acid. Interestingly, the growth rate and yields decreased with increasing oleic acid concentration, most likely due to an inhibitory compound present in the olive oil.

### Testing Optimized Media in Micro-Fermentations (BioLector^®^) vs. Lab Scale Bioreactors

The knowledge obtained in the screening of media components was combined in order to design an optimal medium composition. CBS-glycerol medium with a C/N ratio of 0.875 (glycerol: 20 g/L, (NH_4_)_2_SO_4_: 40 g/L) was chosen as the basis, pH was either 7 or 8, and oleic acid was added (1%) or left out. As seen in Figure 2F, the highest specific growth rate was observed for pH 7 and 0% oleic acid, whereas the biomass concentration, β-carotene concentration, and Y_SX_ were higher at pH 7 with 1% oleic acid. Based on these results, we decided to move forward with these two media formulations for the scale up to 1L bioreactors. Both the biomass and β-carotene formation seemed to be impaired at pH 8, most likely due to a prolonged alkaline stress.

In order to determine the predictability of the observed results when scaling-up to 1L, four sets of conditions from the micro-fermentations were chosen, namely the two optimized media compositions: CBS-glycerol pH 7, C/N 0.875 (CBS-gly-opt%OA) and CBS-glycerol pH 7, C/N 0.875, 1% oleic acid (CBS-gly-opt + OA), along with CBS-glycerol pH 5 C/N 7, and CBS-glucose pH 5 C/N 7.3. The two optimized media compositions were chosen as they provided the best results. CBS-gly pH 5 C/N 7 was chosen as promising results had been observed too. The last set of conditions (CBS-glc pH 5 C/N 7.3) was chosen as the performance in the micro-fermentation set-up was very poor, therefore it was predicted that this would also occur in 1L scale. Furthermore, both experiments at C/N ∼7 were also chosen to illustrate and investigate the differences between carbon sources.

**FIGURE 3:**
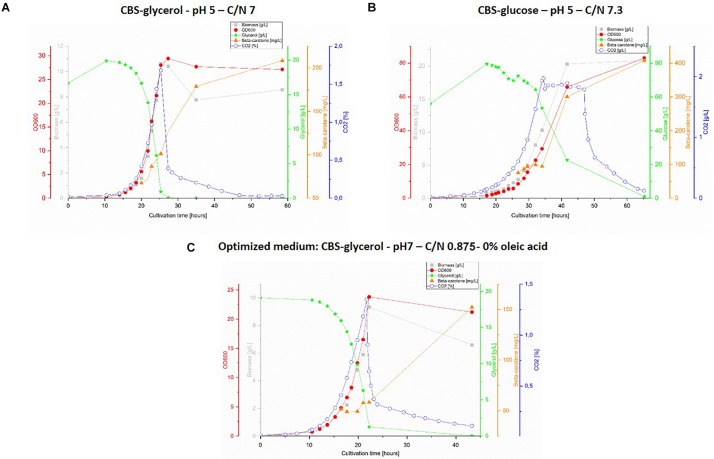
1L bioreactor fermentations. Top panel: cultivation with CBS-glycerol medium, pH 5 and C/N 7 **(A)** and cultivation with CBS-glucose, pH 5 and C/N 7.3 **(B)**. Lower panel **(C)**: cultivation with optimized medium: CBS-glycerol, pH 7, C/N: 0.875 and 0% oleic acid. The graphs show representative replicate from triplicate series.

The obtained results are presented in Figure 3, however, no data is shown for CBS-gly-opt + OA as the design proved unfit for bioreactor cultivation due to severe foaming, which could not be controlled by adding antifoaming agent. Very little difference could be observed between the graphs for the two glycerol-containing media (Figures 3A,C). The substrate consumption and the specific growth rate were slightly higher for CBS-gly pH 5 C/N 7; 0.300 ± 0.037 h^–1^ vs. 0.282 ± 0.025 h^–1^ for CBS-gly-opt%OA, whereas the lag-phase was shorter for CBS-gly-opt%OA (approx. 8 h) than CBS-gly pH 5 C/N 7 (approx. 12). Despite almost identical C/N ratios, CBS-glc pH 5 C/N 7.3 (Figure 3B) showed very different growth kinetics to CBS-gly pH 5 C/N 7 (Figure 3A). The lag-phase of CBS-glc pH 5 C/N 7.3 was the longest in the set (15 h) and the slopes of biomass, OD_600_ and CO_2_ were the lowest, which resulted in an overall growth rate of 0.184 ± 0.035 h^–1^. A plateau was also seen in CO_2_-production and the longer lag-phase once again confirmed that glucose was not the preferred substrate of *Y. lipolytica* ob-CHC^TEF^C^TEF^. In addition, the lower slope in the exponential phase was partly an evidence of this, and it also proved the inhibitory effect reported at high initial carbon-concentrations.

### Correlation Between Micro-Scale and Lab-Scale Fermentations

In order to compare the results obtained in micro-fermentations and 1L bioreactors, the data was collected and compared for all six optimization parameters (Table 1). It was found that in micro-fermentations, the specific growth rate was underestimated compared to the actual value found in the bioreactor experiments. The specific growth rates from bioreactors had been calculated from more data-points obtained using three different methods: OD, DW, and accumulated CO_2_. In the case of the micro-fermentations, it was only possible to calculate specific growth rates from LSU due to the volume limitation. Therefore, the specific growth rates from bioreactors are more reliable. For the glycerol-containing media designs, the biomass concentration was accurately estimated in the micro-fermentations when compared to the bioreactors. In contrast to the glucose-containing medium, CBS-glc C/N 7.3, where the amounts of biomass and β-carotene were dramatically different from the ones obtained in the 1L scale. This could be explained by the differences in cultivation times and the potentially remaining carbon source in micro-scale that was completely consumed in the bioreactor. Quantifying carbon-source in the micro-fermentations was not possible due to low working volumes. The concentration of β-carotene was also overestimated in CBS-gly-opt%OA and CBS-gly pH 5 C/N 7 micro-fermentations to greater or lesser extent. Estimated yields follow the same trends, as they are calculated from the abovementioned parameters, thus even small variations in β-carotene concentrations between the micro-and lab-scale fermentation systems would lead to larger variations in yields.

**TABLE 1:** Key numbers for comparison of micro-fermentations and lab-scale fermentations.

	**CBS optimized wo. Oleic acid *CBS-gly-opt%OA* C/N ratio: 0.875 pH: 7 Glycerol: 20 g/L (NH_4_)_2_SO_4_: 40 g/L**		**CBS-glycerol - Baseline C/N ratio: 7 pH: 5 Glycerol: 20 g/L (NH_4_)_2_SO_4_: 5 g/L**		**CBS-glucose C/N ratio: 7.3 pH: 5 Glucose: 80 g/L (NH_4_)_2_SO_4_: 5 g/L**		**YPD-glucose Initial medium: Yeast extract: 20 g/L Peptone: 40 g/L Glucose: 5 g/L Feed: 6g/h glucose**
				
	**1L Bioreactor**	**Microferm.**	**1L Bioreactor**	**Microferm.**	**1L Bioreactor**	**Microferm.**	**5L Fed-batch Larroude et al. (2017)**
Specific growth rate (μ) [h^–1^]	0.282 ± 0.025	0.239 ± 0.008	0.300 ± 0.037	0.229 ± 0.003	0.184 ± 0.035	0.121 ± 0.0.25	–
Biomass conc. [g/L]*	6.933	7.811	7.568	6.789	20.812	7.285	72
Yield (Ysx) [gDW/gSubstrate]	0.369	0.391	0.381	0.340	0.262	0.092	0.080
Beta-carotene conc. [mg/L]	151.141	416.63	208.475	282.89	407.958	53.15	6500**
Yield (Ysp) [gBC/gSubstrate]	0.008	0.021	0.010	0.014	0.050	0.001	0.007
Yield (Yxp) [gBC/gDW]	0.022	0.053	0.028	0.042	0.020	0.007	0.09

Data is presented for three media types from this study and fed-batch data from Larroude et al. (2017). Specific growth rates and biomass concentration have been calculated from triplicates. Yields and β-carotene has been determined from representative replicates. * all standard deviations were below 1.144 g/L. ** note that in this case, the final concentration is provided at the end of a fed-batch process, therefore with a constant rate of glucose addition of 6 grams per hour throughout the run.

The data obtained showed that the general trends observed in the BioLector^®^ were similar to the ones observed in standard 1L bioreactors. The two glycerol-containing media compositions performed well in the screening and the test of optimized medium in small scale. The results from the 1L scale showed that they also performed equally well there, whereas CBS-glc C/N 7.3 performed poorer in both scales, as originally hypothesized. It is worth mentioning that of course absolute values may differ when comparing a micro-fermentation system to lab-scale bioreactors, due to differences in kinetics, volume and analysis methods. However, the improvements obtained through experimentation at micro-scale, follow the same tendency in lab-scale reactors when running the same experiments. In our work, the ranking of experiments from best to worst in micro-scale translated directly into the same ranking at 1L lab-scale. The predictability from micro-scale to lab-scale proves the worth of using micro-fermentors in the design and optimization of bioprocesses, as the number of conditions to be tested at lab-scale can be reduced to only include the best candidates. The comparison proved to also be robust despite of the differences in inoculum size and cultivation time required by the different systems.

**FIGURE 4:**
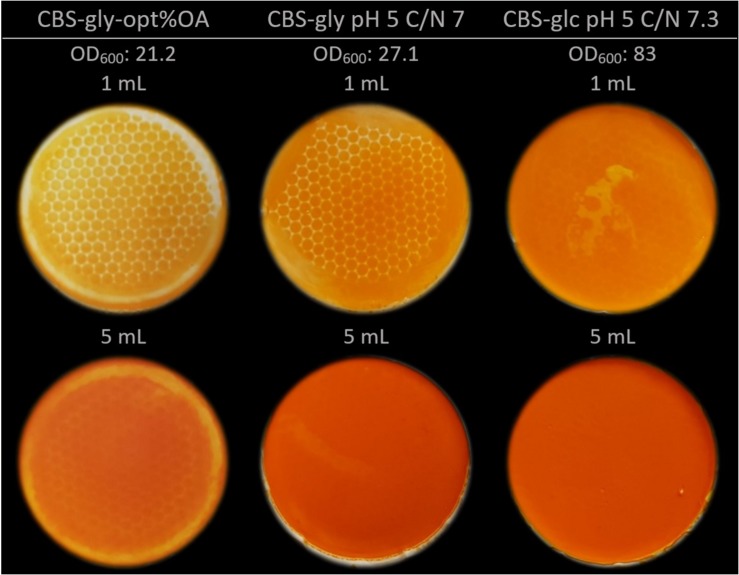
Color intensity of fermentation broth from three different media. Top panel: 1 mL of crude fermentation broth filtered through 0.45 μm filters and washed. Bottom panel: 5 mL of crude fermentation broth filtered through 0.45 μm filters and washed. NB: please notice difference in OD_600_.

To visually assess the β-carotene content in the fermentation broth, pictures were taken of filtered samples from the bioreactor experiments (Figure 4). Both the two media with C/N 7, namely CBS-gly pH 5 C/N 7 and CBS-glc C/N 7.3, exhibit an intense orange color which appears almost red in the bottom panel where 5 mL were filtered. It should be considered that the cell density for CBS-glc C/N 7.3 was three times higher than that of CBS-gly C/N 7. Both visually and based on numbers, glycerol is the best carbon-source for β-carotene production. The samples of CBS-gly-opt%OA appear less pigmented even if one considers the difference in cell density.

### The Strength of Using Micro-Fermentations in Cell Factory Design and Testing

As the data obtained in microscale can successfully predict the results to be seen in larger scale, micro-fermentations can definitely be considered a powerful tool, not only for the bioprocess design itself but also in the process of cell factory design. Considering the design-build-test-learn cycle (DBTL-cycle), described by Frost et al. (2018), it will be possible to shorten the *“test”* phase if robust and easy-to-use high-throughput systems are available. As the level of predictability is high for micro-fermentations, it would be possible to only use those in the first iterations of the cycle, before scaling the process to a larger scale.

In this study, the data generated at micro-scale did also provide some knowledge on the impact of the cultivation conditions at the cellular level, which could become useful for future strain engineering. The strain *Y. lipolytica* CHC^TEF^C^TEF^ was designed to accumulate more lipids by engineering of the *de novo* pathway, along with blocking of the degradative pathway for storage lipids. The original paper showed a positive correlation between the aforementioned strategy and increased β-carotene synthesis (Larroude et al., 2017).

In our work, the *de novo* synthesis along with the incorporation of extracellular lipids from the medium, was further investigated in order to optimize β-carotene production. Interestingly, our results suggest that the lipid accumulation could be negatively correlated to β-carotene synthesis, as β-carotene yields were lower when the lipogenesis was stimulated. This finding could indicate that the importance is not related to the lipids themselves but rather to the metabolic regulation of pathways within the cell. Moreover, it is possible that a specific level of lipid accumulation is optimal for β-carotene synthesis, and that levels below and above this threshold could lead to a reduction in product formation. These results and speculations could therefore guide a second round of the design-build-test-learn cycle to further improve strain performance and optimize the medium.

## Conclusion

Based on the presented results, we conclude that micro-fermentations represent a powerful tool which should be taken into consideration within the field of cell factory design. They provide accurate, robust and scalable results that can be used for screening and characterization of both newly discovered organisms and engineered strains. The use of micro-fermentations can dramatically reduce the duration of the *“test”*-phase in the DBTL-cycle without losing fundamental information. The workload can also be dramatically decreased, as very little user-input is needed during the cultivation. As suggested by our data, micro-fermentations can provide knowledge on unexpected changes in metabolism, which could prove problematic later in the cell factory construction, characterization, and perhaps establishment of a larger-scale production.

In terms of bioprocess design and optimization, our study also shows that micro-fermentation setups represent a good strategy for setting a strong baseline, which can serve as a reliable starting point for designing a suitable production strategy in larger scales, e.g., further fed-batch design at pre-pilot level prior to industrial production levels. This would allow, on one hand, to reduce the costs in terms of resources spent in the process design strategy (media composition and optimal cultivation conditions), and on the other hand significantly decreasing the time-frame for this stage, as multiple conditions and replicates can be tested in one run, hence again contributing to a faster DBTL-cycle therefore speeding up the transition from lab to industrial process.

## Data Availability Statement

The datasets during and/or generated during the current study available from the corresponding author on reasonable request.

## Author Contributions

IJ, RL-A, and JM conceptualized and created the framework for the study. IJ performed the experiments and data analysis. RL-A and JM supervised the work. IJ, RL-A, and JM wrote and edited the manuscript.

## Conflict of Interest

The authors declare that the research was conducted in the absence of any commercial or financial relationships that could be construed as a potential conflict of interest.
